# Decidualized ovarian endometrioma mimicking malignancy in pregnancy: a case report and literature review

**DOI:** 10.1186/s13048-022-00966-6

**Published:** 2022-03-09

**Authors:** Min Yin, Tao Wang, Sijian Li, Xinyue Zhang, Jiaxin Yang

**Affiliations:** 1 Department of Obstetrics and Gynecology, Peking Union Medical College Hospital, Chinese Academy of Medical Sciences & Peking Union Medical College, Beijing, China; 2 National Clinical Research Center for Obstetric & Gynecologic Diseases, Beijing, China

**Keywords:** Decidualized endometrioma, Pregnancy, Ultrasound, Magnetic resonance imaging

## Abstract

**Background:**

Ovarian endometrioma is a common gynecologic disease among reproductive-aged women. Pregnancy-related hormonal status may lead to changes of decidualization, which may resemble ovarian malignancies in sonographic appearance. Here we present a case of decidualized ovarian endometrioma clinically mimicking malignant transformation.

**Case presentation:**

A 37-year-old pregnant woman presented to our hospital at 25 + 5 weeks of gestation with a persistent left adnexal mass that was first detected on routine ultrasound in the first trimester. Transvaginal and transabdominal ultrasound showed a cystic mass of size 8.4 × 5.8 cm in the left ovary with abundant blood flow signals in the papillary medium echo of the capsule wall and inner wall, raising concern for malignant ovarian tumor. After a multidisciplinary discussion, the patient underwent laparoscopic left salpingo-oophorectomy. The results of the frozen section revealed decidualized endometrioma and the final histopathology confirmed endometrioma with extensive decidualization. The patient’s postoperative recovery was uneventful and she was discharged on the 4th postoperative day.

**Conclusions:**

Decidualized ovarian endometrioma is rare. Sonography and magnetic resonance imaging are helpful for differential diagnosis. Conservative management of expectant management and serial monitoring should be adopted if decidualized endometriosis is suspected.

## Background

Adnexal mass is the most common indication for gynecologic surgery during pregnancy, occurring in 0.1 to 2.4% of pregnant women [1]. Most adnexal masses discovered in pregnant women are unexpectedly found during routine prenatal ultrasonography. The treatment of an adnexal mass is generally conservative because that most are physiologic or benign tumors. In contrast, surgical intervention is required when the mass is suspicious of potential malignancy or severe abdominal pain caused by mass rupture and torsion. Endometriosis is the presence of ectopic endometrial glands and stroma outside the confines of the uterine endometrium [2]. Ovarian endometriomas are a common gynecologic disease among reproductive-aged women. Although ovarian endometrioma with typical traits is easily diagnosed under ultrasonography, changes will occur during pregnancy due to great alterations of the hormone. During pregnancy, the ectopic endometrium takes the change of increasing glandular epithelial secretion, stromal vascularity, and edema under progesterone action, which is defined as decidualization [3]. Decidualized endometrioma with increased blood flow and with intraluminal papillary vegetations results in difficulties in the differential diagnosis from the malignant ovarian tumor [4]. Here we present a case whose decidualized ovarian endometrioma clinically mimicked malignant ovarian tumor.

## Case presentation

**Fig. 1:**
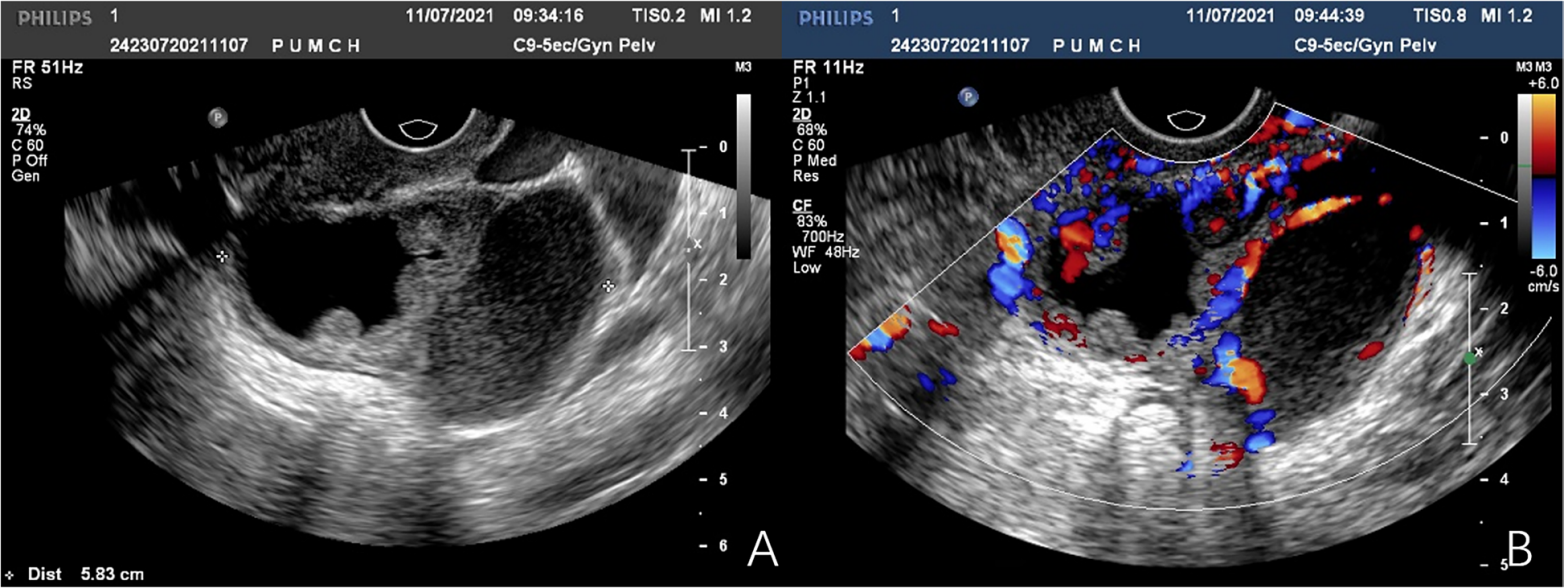
Transvaginal ultrasound imaging of the left adnexal mass. **A** Transvaginal ultrasound depicting multilocular cyst with thick internal walls and papillary projection protruding into the cavity. **B** Doppler ultrasound depicting vascularity within capsule wall and papillary projections

A 37-year-old pregnant woman, gravida 2, para 1, without any systemic disease, was referred to our hospital at 25 + 5 weeks of gestation to evaluate a persistent and growing left adnexal mass found by the first-trimester routine ultrasound. No other obstetric abnormalities were detected in regular antenatal examinations. A left ovarian cyst, 3.4 cm × 2.5 cm, was noted by an ultrasound scan at 7 weeks of gestation. Follow-up sonography showed moderate echogenic protrusions in the ovarian cysts, growing gradually. At the presentation to our hospital, a transvaginal/transabdominal ultrasound was repeated, showing a cystic mass of size 8.4 × 5.8 cm in the left ovary with abundant blood flow signals in the papillary medium echo of the capsule wall and inner wall, which raised concern for malignant ovarian tumor (Fig. 1A and B). Her serum level of CA125 was 44.9 U/mL (normal range, 0–35 U/mL) and CA199 was 41.4 U/mL (normal range, 0–34 U/mL).

**Fig. 2:**
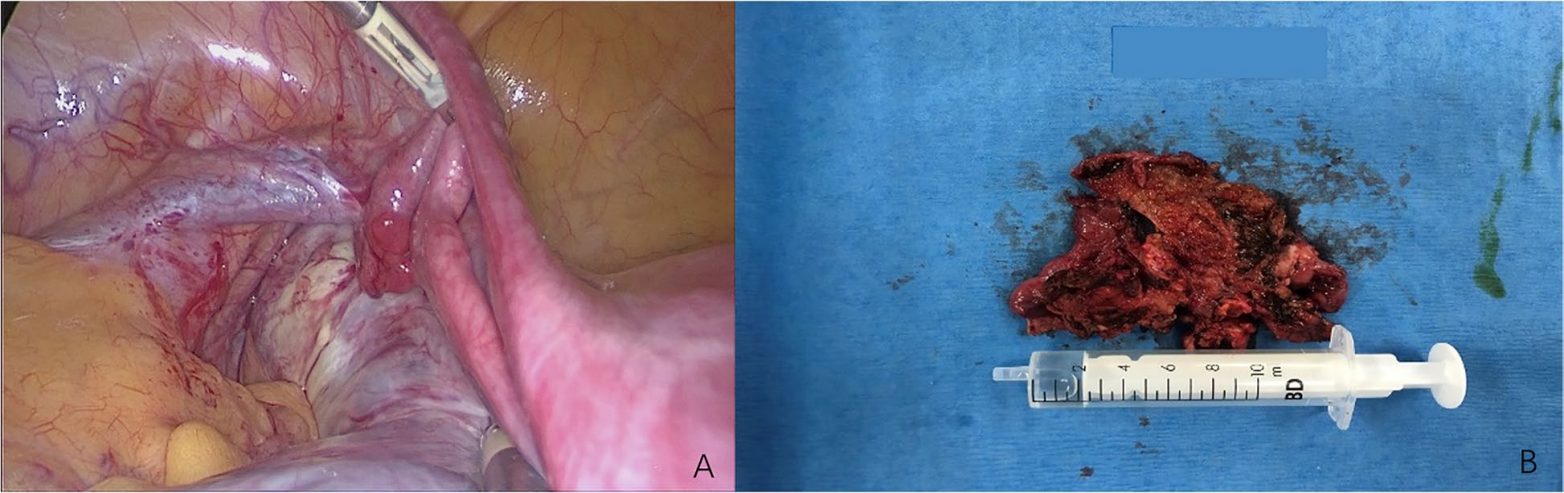
Surgical depiction of the left adnexal mass. **A** Laparoscopy showed an 8 cm left ovarian multilocular cyst adhering to the left pelvic wall and mesorectum. **B** Surgical specimen with brown cystic content and irregular and rounded internal cyst wall

**Fig. 3:**
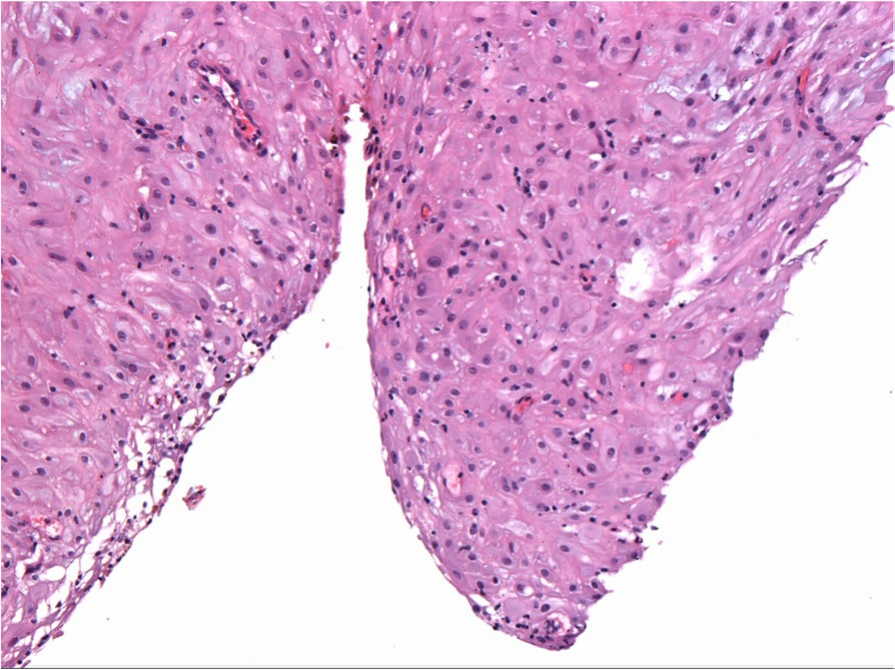
Ovarian endometriosis with extensive stromal decidualization (Hematoxylin and eosin, ×200)

After a multidisciplinary discussion with doctors from obstetrics, gynecology oncology, and ultrasound departments, it was considered that the left adnexal mass was likely to be ovarian malignancy. Compare with ovarian cystectomy, salpingo-oophorectomy can effectively reduce the incidence of tumor rupture, therefore avoiding the intraperitoneal dissemination of tumor cells and avoiding the increased clinical stage caused by the operation as much as possible. Based on the above considerations, laparoscopic left salpingo-oophorectomy was recommended instead of ovarian cystectomy. After fully informed consent, the patient chose to undergo left salpingo-oophorectomy at 26 + 1 weeks of gestation. It was observed that an 8 cm left ovarian multilocular cyst can be seen and the cyst adhered to the left pelvic wall and mesorectum (Fig. 2A). Adhesion lysis was performed and the cyst ruptured during manipulation. Brown cystic content and irregular and rounded internal cyst wall were observed. After left salpingo-oophorectomy, we did careful examination if the specimens: the left ovarian cyst is multilocular, and the inner wall of the cyst is thickened and villous (Fig. 2B). The results of the frozen section showed decidualized ovarian endometriomas, and the final histopathology confirmed endometrioma with extensive decidualization (Fig. 3). The patient’s postoperative recovery was uneventful, and the fetal heart rate was detected before and after surgery to evaluate the fetal status. She was discharged on the 4th postoperative day.

## Discussion

Adnexal masses during pregnancy are problematic because of the increased difficulty of surgery and the risk of abortion and preterm birth. The incidence of adnexal masses during pregnancy varied, 11% of which are endometriomas and the reported incidence of ovarian malignancy is only 1% [5]. During pregnancy, normal endometrium will convert into a specialized uterine lining adequate for optimal accommodation of the pregnancy, which is called decidualization. Under the action of progesterone, hypertrophy of the endometrial stromal cells leads to cyst enlargement and irregular and vascular solid components, mimicking malignancy on imaging, potentially leading to unnecessary surgical intervention. Decidualized ovarian endometrioma in pregnancy is a rare condition and we only identify 18 studies comprising a total of 61 cases of ovarian decidualized endometriomas during pregnancy after the literature review [3, 4, 6–21]. The main characteristics of the published cases are summarized in Table 1.

**Table 1:** Summary of reported decidualized ovarian endometrioma cases during pregnancy

Author, reference	Publication year	No. of cases	Age, range	CA125 (U/ml)	Laterality	Size (cm)	Solid part (cm)	Intracystic papillae	Septa	Blood flow	MRI	Surgery GA	Surgical procedure
Miyakoshi K et al. [6]	1998	1	28	NR	Unilateral	Dmax 8.5	NR	+	–	+	+	20	Oophorectomy
Tanaka YO et al. [7]	2002	1	27	103	Unilateral	12 × 8 × 7	NR	+	+	NR	+	12	Cystectomy
Fruscella E et al. [8]	2004	1	39	76	Unilateral	Dmax 5.5	0.8 × 1.0 and 0.5 × 0.5	+	–	+	+	18	Oophorectomy
Sammour RN et al. [4]	2005	2	36	NR	Unilateral	3.7 × 2.7 × 3.5	1.5 × 2.0 × 2.5	+	–	+	+	15	Oophorectomy
			28	NR	Unilateral	4.0 × 5.0 × 6.3	2.3 × 1.8 × 1.4	+	–	+	–	16	Oophorectomy
Guerriero S et al. [9]	2005	1	38	109	Bilateral	4.0 × 4.8	NR	+	–	+	–	37 (CS)	Bilateral Cystectomy
Iwamoto H et al. [10]	2006	1	31	28.3	Unilateral	Dmax 7.5	NR	+	–	+	+	22	Oophorectomy
Asch E et al. [11]	2007	1	NR	NR	Unilateral	NR	NR	+	–	+	–	After delivery	Bilateral cystectomy
Poder L et al. [12]	2008	1	34	24	Unilateral	Dmax 6.2	+	+	–	+	+	38 (CS)	Oophorectomy
Machida S et al. [13]	2008	3	32	119	Unilateral	8 × 5	NR	+	–	+	+	19	Oophorectomy
			41	220	Unilateral	Dmax 16	NR	+	+	NR	+	14	Oophorectomy
			24	34	Bilateral	Dmax 5	NR	+	–	NR	+	14	Cystectomy
Yoshida S et al. [14]	2008	2	29	NR	Unilateral	8.5 × 5.3	NR	+	–	–	+	14	Oophorectomy
			28	NR	Unilateral	7.4 × 4.8	NR	+	–	–	+	19	Oophorectomy
Takeuchi M et al. [15]	2008	5	20–32	NR	Unilateral	4–9	2 (max)	+	–	–	–	NR	NR
Barbieri M et al. [16]	2009	3	32	30	Unilateral	6.6 × 4.4	2 × 1.4	+	–	+	–	NR	Cystectomy
			36	159	Bilateral	8.5 × 6.3, 5 × 3	2.7 × 1.4,0.7 × 0.7	+	–	+	–		None
			39	85	Unilateral	3.8 × 1.4	1.9 × 1.2	+	–	+	–		None
Sayasneh A et al. [17]	2012	1	35	89	Unilateral	3 × 4 × 4	1.5	+	–	+	–		None
Tazegül A et al. [3]	2013	1	32	220	Unilateral	6.5 × 5.7	1.4 × 0.8	+	–	+	–	12	Cystectomy
Proulx F et al. [18]	2014	1	30	NR	Unilateral	Dmax 4	NR	+	–	+	+		None
Mascilini F et al. [19]	2014	18	20–43	12–285	3 bilateral and 15 unilateral	4.1–12.1	17 cases +	17 cases +	7 cases +	16 cases +	–		13 cases
Groszmann Y et al. [20]	2014	17	22–43	NR	5 bilateral and 12 unilateral	3–27	14 cases +	NR	8 cases +	12 cases +	–		8 cases
Alsalem H et al. [21]	2021	1	28	134	Unilateral	NR	+	+	–	+	+	38 (CS)	Cystectomy

*Abbreviations*: *MRI* magnetic resonance imaging, *GA* gestational age, *NR* not reported, *Dmax* maximum diameter, *CS* Cesarean section

Surgeries during pregnancy may be avoided or at least delayed until the term or postpartum period if diagnostic accuracy can be improved. Sonography is regarded as the gold standard imaging method for the diagnosis of ovarian endometriomas in non-pregnant women, with the reported sensitivity and specificity to be 84–100% and 90–100%, respectively [15]. The typical feature of an ovarian endometrioma is a round-shaped cystic mass with regular margins, homogeneous low echogenic fluid content with scattered internal echoes, and without papillary proliferations. Several case reports have shown the sonographic findings of decidualized endometriomas with septations, nodularity, and marked blood flow, which are similar to those that may be seen in borderline or malignant tumors, posing a challenging diagnostic dilemma. Barbieri M et al. [16] reported three cases with an ovarian endometrioma in pregnancy mimicking malignancy and performed a literature review from 1990 to 2008. They found that rapidly growing and vascularized intracystic excrescences were consistently documented, but septations or significant free fluid was never reported. What’s more, they reported an interesting case suggesting that decidualized endometrioma is a transitory transformation.

Mascilini F et al. [19] retrospectively reviewed 18 women with a histological diagnosis of decidualized endometrioma during pregnancy from seven ultrasound centers. They found that 14 of the 17 decidualized endometriomas manifested vascularized rounded papillary projections with a smooth contour and ground-glass or low-level echogenicity of the cyst fluid when using pattern recognition. Therefore, they suggested expectant management of cysts with papillary projections detected during pregnancy if an endometrioma was diagnosed before pregnancy and manifests the typical signs of decidualized endometrioma. Furthermore, Groszmann Y et al. [20] described the sonographic characteristics of 22 decidualized endometriomas in 17 patients. They used the International Ovarian Tumor Analysis Group definitions for adnexal masses but failed to identify characteristic sonographic features to distinguish decidualized endometrioma from ovarian malignancy. However, they suggested delaying surgery until delivery or postpartum if the mass showed no change in size over 4 weeks or lacked solid components and vascularity. Recently, Alsalem H et al. [21] reported a case with a growing left adnexal mass and elevated CA125 at 134kU/L. After a transvaginal/transabdominal ultrasound, the possibility of a benign decidualized endometrioma was raised and the patient consented to an ovarian cystectomy at her cesarean section, preventing an unnecessary oophorectomy during pregnancy and reducing the anxiety of malignancy. Finally, the histopathology confirmed endometriosis with extensive decidualization and no malignancy.

Gadolinium-free magnetic resonance imaging (MRI) during pregnancy is considered safe and can be used to evaluate ultrasound-undetermined adnexal lesions. Because of the vascularization and edema, the apparent diffusion coefficient (ADC) of decidualized endometrial tissues was significantly higher than that of ovarian cancers [15]. Nobuko Morisawa et al. [22] investigate the MRI findings of 18 decidualized endometriotic cysts in comparison with 24 endometriotic cysts associated with ovarian cancers. They found that the heights of the solid components in the decidualized endometrioma were significantly lower compared with the ovarian cancers, and solid component showing high signal intensities on T2-weighted imaging is highly indicative of decidualization. Recently, Takeuchi et al. [23] evaluated the diagnostic ability of computed diffusion-weighted imaging (DWI) for differentiating decidualized endometrioma from ovarian cancer. In this study, mural nodules in decidualized endometriomas showed high signal intensity on DWI with b values of 800 s/mm^2^ due to T2 shine-through effect. However, the decidualized endometriomas showed a signal decrease on computed DWI with b values of 1500 s/mm^2^, whereas cancers did not. Hence, computed DWI with b values of 1500 s/mm^2^ may be useful to distinguish decidualized endometriomas from ovarian malignancy. Overall, ADC measurement and DWI with high b values are recommended as additional diagnostic tools.

As for serum tumor markers, some studies indicated that CA125 levels have a limited value of differential diagnosis in pregnant patients because it is physiologically elevated [24]. However, some studies have proposed a potential diagnostic role of continuous measurement of CA125 or the levels of CA125 above 1000 U/ml at or after mid-pregnancy [25]. Moreover, the serum concentration of human epididymis protein 4 (HE4) marker was reported to increase with the duration of pregnancy. Gasiorowska E et al. [26] demonstrated that the upper limit values of normal HE4 levels are 55 pmol/l, 80 pmol/l, and 106 pmol/l for the first, second, and third trimesters, respectively. Therefore, the combined evaluation of CA125 and HE4 may play a potential role in the differential diagnosis of adnexal masses during pregnancy.

Actually, it is a dilemma between expectant treatment and surgical intervention concerning the management of adnexal masses during pregnancy. This may lead to unnecessary resection of benign tumors, or even worse, conservative observation of a malignant ovarian tumor. Therefore, it is necessary to hold a multidisciplinary discussion to balance the fetal and maternal risks, gestational age, and degree of malignant suspicion. It is worth noting that, according to published literature, the pregnancy outcome of patients undergoing surgery is safe, only one patient suffered a preterm rupture of membranes on the day of laparotomy at the 19th gestational week [13]. Among the published literature shown in Table 1, all patients who chose to surgery underwent laparotomy. Nowadays with the development of laparoscopic techniques, laparoscopy performed by an experienced surgeon has been reported to be a safe approach during pregnancy [27]. In our case, laparoscopic left salpingo-oophorectomy was performed and the patient recovered smoothly without postoperative complications.

## Conclusion

Pregnancy-related changes of ovarian endometrioma leading to the vascularized intracapsular vegetation mimicking malignancies are rare but possible events. In this case, we reported a decidualized ovarian endometrioma mimicking malignant transformation during pregnancy. Sonographic findings and the use of MRI are helpful for differential diagnosis. Conservative management of expectant management and serial monitoring should be adopted if decidualized endometriosis is suspected.

## Data Availability

All the generated data are included in this article.

## Abbreviations

MRI: Magnetic resonance imaging
ADC: Apparent diffusion coefficient
DWI: Diffusion-weighted imaging
HE4: Human epididymis protein 4
